# Perception of the local community: What is their relationship with environmental quality indicators of reservoirs?

**DOI:** 10.1371/journal.pone.0261945

**Published:** 2022-01-21

**Authors:** Evaldo de Lira Azevêdo, Rômulo Romeu Nóbrega Alves, Thelma Lúcia Pereira Dias, Érica Luana Ferreira Álvaro, José Etham de Lucena Barbosa, Joseline Molozzi

**Affiliations:** 1 Biological Sciences Coordination, Institute Federal de Educação, Ciência e Tecnologia da Paraíba, Princesa Isabel, Paraíba, Brazil; 2 Department of Biology, University Estadual da Paraíba, Campina Grande, Paraíba, Brazil; Universitat de Barcelona, SPAIN

## Abstract

Evaluating the conservation of aquatic ecosystems, especially those which serve to supply, has been carried out using a variety of tools. However, the perception of water quality by the local community which lives in direct contact with water resources has not been considered with enough importance. This study analysed the relationship between the conservation status of reservoirs as perceived by the local community and their conservation status according to physical, chemical, and biological indicators. To do so, we calculated the Trophic State Index (TSI) of the reservoirs, the diversity and richness of benthic macroinvertebrate and we analysed the human influence in the riparian zone. Thus, we created the Community Conservation Perception Index (CCPI) to quantitatively associate the perception of the local community with environmental quality indicators (TSI, diversity, richness and anthropogenic influences in the riparian zone). We found that interviewee perception of reservoir conservation (using the CCPI) was related to trophic state, richness and diversity of benthic macroinvertebrate, and the presence of residences and agriculture in the riparian zone. It is necessary to consider the environmental perception of the local community as a relevant factor in management programmes and the conservation of ecosystems, even if artificial, as is the case with reservoirs. These communities can significantly contribute to maintaining the environmental quality through their performance in participatory management in projects such as: participating in the investigation of pollution in reservoirs, collecting parameters related to water quality, and community action in designing conservation strategies.

## 1. Introduction

Different aquatic ecosystem monitoring methodologies have been implemented in several parts of the world, for example: the Water Framework Directive [1]; United States Environmental Protection Agency (US EPA) [2]; and CONAMA Resolution 430/Brazil [3]. Many researchers have searched for holistic evaluation methods based on integrating different monitoring tools [4–10]. However, the integration of interactions and perceptions of human beings has not been routinely implemented in monitoring programmes, even though it has been deemed necessary for many years [11].

The most frequently used tools considering the different environmental quality evaluation forms of aquatic ecosystems are bioindicators [6, 12–15], environmental variables [16, 17] and landscapes [5, 18]. Benthic macroinvertebrates stand out among water quality bioindicators (such as macrophytes, benthic macroinvertebrates, phytoplankton, zooplankton, and fish) due to their ability to reflect environmental alterations over a long-term period through changes in structure and distribution [6, 19, 20], and therefore they were chosen as biological indicators in this study. Oligochaetes, Mollusca and Diptera (Chironomidae) are the most represented taxa in Brazilian reservoirs [21]. Changes in water-related environmental variables tend to influence the distribution, richness and diversity of these taxa.

When considering environmental variables, it is important to assess trophic state, as it reflects anthropogenic influence on water quality and the ecological functioning of aquatic ecosystems, providing a summarised vision of organic enrichment, water transparency and phytoplankton development [22]. Several studies have indicated that the Trophic State Index is an efficient tool [23–27]. Riparian zone analysis is also important to evaluate water bodies. Alterations in riparian zone due to urbanisation, agricultural and industrial activities [18, 28] can affect biodiversity, increase soil erosion, modify ecosystem services, intensify natural disasters, affect water quality and harm socio-cultural practices [29–31].

The legislation in Brazil provides for the decentralized management of watersheds through the institution Hydrographic Basin Committees through the law 9433/1997 [32], which establishes the National Water Resources Policy and Resolution No. 5/2000 of the National Water Resources Council (CNRH) [33], which in turn establishes guidelines for the functioning of the Hydrographic Basin Committees. One of the main responsibilities of these committees is to approve the basin’s Water Resources Plan, a document that defines actions such as water use, recovery, protection, and water resource conservation. It is composed of representatives of the Union, States, Federal Districts, Municipalities, water users in the basin, and the civil entities with proven performances in the basin. However, there are some difficulties associated with the participation of local community representatives in these committees, in addition to existing conflicts, mainly in relation to the use and management of water resources, and consequently the reflection of these relationships (harmonic or disharmonious) in aquatic ecosystems [34–36]. It is important to consider all of these aspects, since information extracted from local communities can offer political and technical insights which are often neglected by professionals [34, 36]. Thus, the development of integrative and participative management programmes is necessary [37], considering the complementation between scientific and local perception in their different phases [35, 38, 39]. In this sense, metrics developed from the perception of the local communities can aid to improve management policies, being used as part of the performances to promote participatory management.

This analysis is important as humans are intrinsically connected to hydrological cycles, since they interact with this resource in several ways such as by collecting water to produce food, obtaining electric energy, drinking water supply, polluting water sources, implementing policies and management technologies [40–42]. Thus, analysing the perception of local communities in terms of resources aids in identifying different water uses [43] or even the impacts affecting ecosystems [23]. It is important to consider that perceptions incorporate cognitive, emotional and cultural factors, as well as personal experience, socioeconomic status, educational level, understanding, experience, proximity and contact with the environment; these factors influence the way people interpret the environment and express their perception about it [37, 44].

It is fundamental that the perception of the local communities regarding the ecosystems they interact in (through water use, fishing, agriculture, among others) [43], in addition to their perception about the conservation of theses ecosystems are understood, in order to provide scientific use of these perceptions in formulating strategic actions for conservation within a context of participatory management [45]. Perception indexes can be useful in directing conservation action and policies, even in artificial ecosystems such as reservoirs. Thus, identifying and quantifying human perceptions can become important to promote the conservation of natural resources [46], especially when these perceptions are analyzed together with biological, physical and chemical indicators of environmental quality. In this work, we consider the environmental quality (physical, chemical and biological conditions of reservoirs) as a representation of their conservation status, i.e. the maximum ecological potential that the reservoirs could reach in the study period [47].

In this context, developing studies which consider the interactions of local communities with ecosystems, especially studies that approach variables which are potentially related to water quality, are extremely necessary [48]. Therefore, this study analysed the relationship between the conservation status of reservoirs as perceived by the local community and their conservation status according to physical, chemical, and biological indicators. We also aimed to develop a Community Conservation Perception Index (CCPI) to enable comparing local community perceptions with environmental quality indicators. Next, we tested the following hypotheses: 1 –The perception of reservoir conservation by local communities around the reservoirs is related to the trophic state of the reservoirs, benthic macroinvertebrate diversity and richness, and anthropogenic influences in the riparian zone; and 2 –Reservoirs which present better environmental quality levels are those where the local communities claim to practice more conservation strategies, constituting actions which can contribute to improving the environmental quality of reservoirs.

## 2. Methods

### 2.1 Study area

The study area is located in the semi-arid region of Brazil, which spans 969,589.4 Km^2^, covering almost the entire north-eastern region of Brazil [49]. The climate is characterised by presenting average annual temperatures above 20°C and average annual precipitation between 280 and 800mm. The evapotranspiration potential is greater than precipitation levels and rainy periods are concentrated in three or four months of the year [50], which make reservoir ecosystems essential for establishing and sustaining human life in the region.

**Fig 1:**
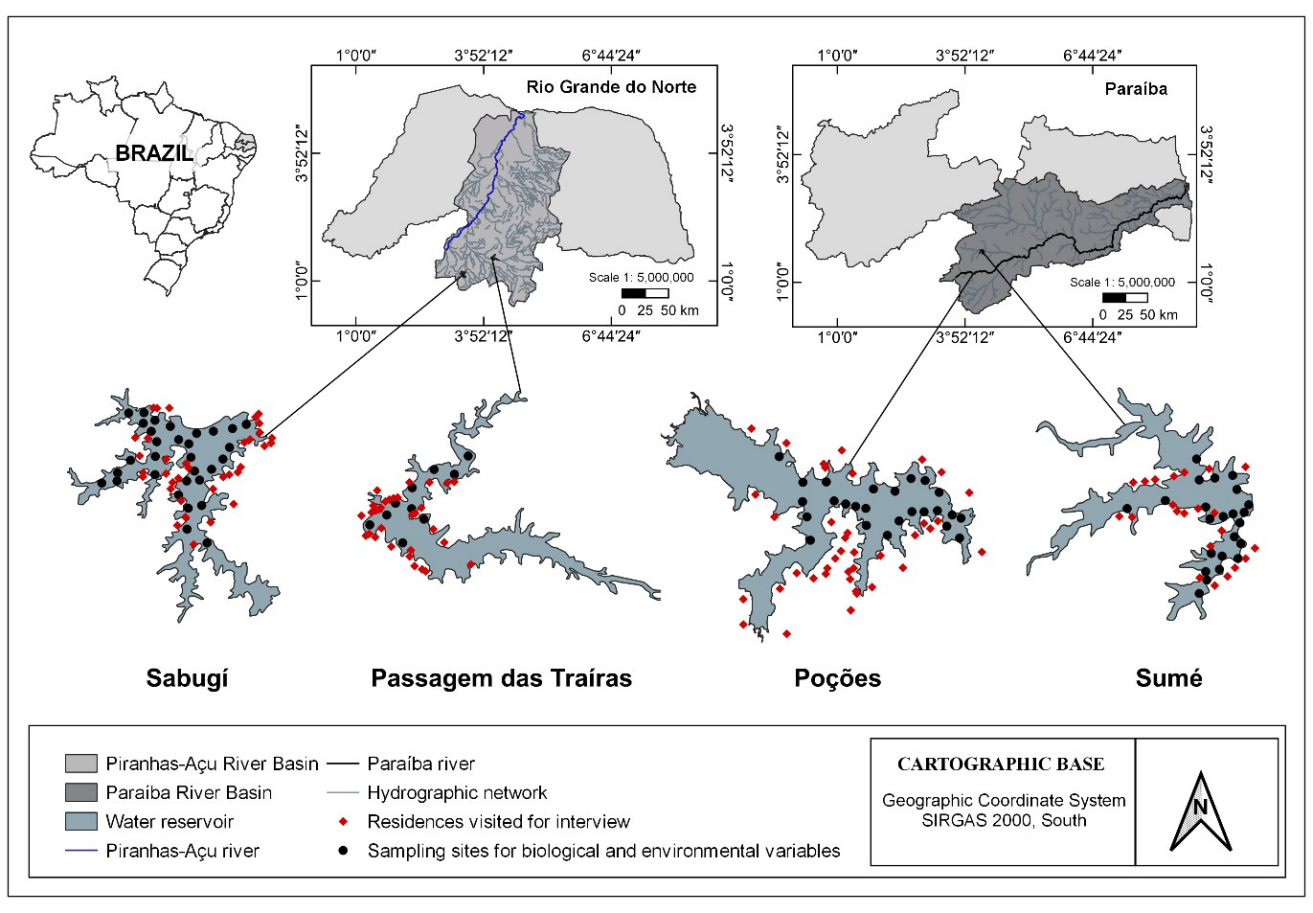
Study area Rio Grande do Norte and Paraíba, communities and reservoirs. Piranhas-Assú River basin and Paraíba River basin. Author: Luciana Marques Rocha Ferreira.

This study was developed in four reservoirs and their surrounding local communities in the north-eastern region of Brazil. Two of the reservoirs were in the state of Paraíba and two in the state of Rio Grande do Norte. The local communities and the Poções reservoir (storage capacity: 29,861,562m^3^) (location: 7°53’33.20" S; 37° 0’31.54" W), and the Sumé reservoir (both in the state of Paraíba) (storage capacity: 44,864,562m^3^) (location: 7°40’14.86” S; 36°54’25.57" W) were studied. Both reservoirs belong to the same hydrographic basin of the Paraíba River. In addition, the communities and the Passagem das Traíras reservoir (storage capacity: 49,702,393.65m^3^) (location: 6°30’52.99" S; 36°55’58.50" W) and the Sabugí reservoir (both in the state of Rio Grande do Norte) (storage capacity: 65,334,880.00m^3^) (location: 6°39’10.79" S; 37°12’20.55" W) were studied, with both reservoirs belonging to the hydrographic basin of the Piranhas-Assú River (Fig 1).

All of the reservoirs studied are primarily used for human supply, have multiple uses such as animal watering, varied domestic uses, agricultural and fishing practices [43]. They are also affected by factors that lead to eutrophication and loss of environmental quality, such as receipt of nutrient loads from urban zones, erosion of the banks, inadequate use and occupation, irrigation, fires, deforestation in the riparian zones, in addition to high water residence time and water stress due to the long drought period [51, 52].

### 2.2 Sample design

#### 2.2.1 Water sampling, macroinvertebrates and evaluation of anthropogenic influences in the riparian zone

The water and macroinvertebrate sampling and the evaluation of anthropogenic influences in the riparian zone of the four reservoirs occurred in four months in 2014 (June, September and December) and 2015 (March). The sampling sites were selected *a priori* in order to represent the environmental variability within each reservoir, as well as the proportion of points in relation to the area of each reservoir. However, some selected locations could not be accessed due to a historical drought that covered the entire study period [53]. A total of 332 samples were collected in the four months for each analyzed item (water, macroinvertebrates, anthropogenic influences in the riparian zone), with 24 points sampled for Poções in June, 21 in Sept., 22 in Dec., and 19 in Mar.; 22 points sampled for Sumé in June, 21 in Sept., 20 in Dec., 23 in Mar.; 10 points sampled for Traíras in June, Sept. and Dec, and 9 in Mar.; and 30 points sampled for Sabugí in June, Sept and Mar., and 31 in Dec.

#### 2.2.2 Sample design and socio-environmental data collection

A total of 126 people were interviewed in the months of September and October 2015, with the majority being males (64.3%, n = 81), while women represented 35.7% (n = 45) of the interviewees. Thus, 38 people were interviewed around the Poções reservoir, 22 in the community of the Sumé reservoir, 31 in Traíras, and 35 in Sabugí. The criterion chosen to select respondents was the individual residing as close as possible to the reservoir. The homes visited were selected using Google Maps. The distance between them to the reservoir margin was measured, which resulted in choosing residences which were at an average distance of 200 m from the reservoir. We assumed that residing closer to the reservoir can result in a greater possibility of interactions with them. The interviews were performed with the head of the family of each residence visited, who was either male or female; they were chosen because of their greater possibility of residing in the locality longer than other younger residents, in addition to generally being providers for the home, which can make them more likely to explore resources and develop more interactions with reservoirs.

A semi-structured form was used to collect socio-economic data, information about the perception of the conservation state of the reservoirs and conservation attitudes adopted by the interviewees. The responses were noted on the form and later transferred to a data sheet. After plotting, the answers to the open questions underwent content analysis and were categorized based on the speech theme, with categories created according to the themes that were addressed in the answers [54].

Characterizing the local community: the ages of the interviewees ranged from 18 to 82 years (mean of 51.1, and standard deviation of ± 14.14). Of the illiterate interviewees, 60.3% (n = 76) did not complete elementary school, 3.2% (n = 4) did not complete high school, 3.2% (n = 4) had completed their secondary education, and 1.6% (n = 2) had completed high school. The majority of interviewees were farmers (70%, n = 88), 16% (n = 20) fishermen, 6.3% (n = 8) home caregivers, and 7.7% (n = 10) public workers. The number of years of the interviewee residing near the reservoirs ranged from 1 to 81 years (mean of 25.80, standard deviation of ± 18.40). The average income was R$735.71 (equivalent to approximately US$236.00 in the period).

### 2.3 Collection of variables related to the environmental quality of reservoirs

All analysed parameters, physical, chemical, and biological indicators, and anthropic influences in the riparian zone sought to assess the environmental quality of the reservoirs, which can be expressed by their maximum biological potential [6, 47].

#### 2.3.1 Water quality of the reservoirs

A water sample and transparency assessment was performed in the littoral zone of each of the reservoirs. Water transparency was evaluated *in situ* through measuring the disappearance of a *Secchi* disc. Water samples were collected in plastic bottles from the coastal zone of the reservoirs at an average depth of 50cm at the water’s edge. The samples were then filtered with Whatman GF/C filters in the laboratory. The concentration of dissolved nutrients was analysed based on Standard Methods for the Examination of Water and Wastewater [55]. The filtered samples were submitted to an analysis of soluble orthophosphate (PO_4_). The aliquots of the non-filtered samples were analysed for total phosphorous (TP) through their digestion with potassium persulphate. Chlorophyl-a (Chlo-a) concentration was determined by extracting it from the 90% acetone pigment.

The trophic classification of the reservoirs was performed through the Trophic State Index proposed by Carlson in 1977 and modified by Toledo and collaborators [56]. Values obtained between 0–44 correspond to oligotrophic reservoirs, between 45–54 to mesotrophic, and values >54 indicate eutrophic environments. Toledo et al. (1983) performed the necessary adaptations to apply the index in subtropical aquatic ecosystems, and Azevêdo et al. [25] showed that this index was efficient for determining the trophic status of reservoirs in the Brazilian semi-arid region. In case of replication of this study, the researchers will be able to perform tests with more suitable Trophic Status indexes for the environments according to the study region.

#### 2.3.2 Biological indicators: Macroinvertebrates

The macroinvertebrate sampling took place in the littoral zone of the reservoir at a depth of 50 cm. The littoral zone was chosen because of its greater abundance, richness and diversity of these organisms [57]. The samples were collected using an Ekman-Birge drag (0.225cm^2^). The collected material was transferred to plastic bags and conserved in 4% formaldehyde. The samples were subsequently washed in the laboratory in sieves of mesh size 500μm and kept in 70% alcohol. The identification procedure was carried out using a stereoscope and taxonomic key [58]. The majority of organisms were identified at the family level; however, all the Chironomidae larvae (Diptera, Insecta) were identified at the genus level [59].

#### 2.3.3 Evaluating the riparian zone of the reservoirs

A protocol (Lake Habitat Survey) was used to assess anthropogenic influences in the riparian zone of the reservoirs; the methodology used is in accordance with Rowan et al. [See 60], also applied with adaptations for Brazilian semi-arid reservoirs [See 61]. A plot of 100 m wide (taking the contour of the reservoir margin edge as reference) x 50 m in length (considering the distance from the observer on the margin to the riparian zone) was analysed at each sampling site. The anthropogenic influences investigated within the plot were residences, electric transmission network, fences, pasture and agricultural zones (any plantation areas). The presence of these influences indicates that the area is subject to human disturbance (even if small, as is the case with the presence of transmission lines). The different types of influences and the proportion with which they occur have an impact on the presumption that the soil is unprotected, resulting in a loss of riparian vegetation and in turn loading particles into the reservoir, which can increase the concentrations of nutrients in the water. Such influences were recorded based on their presence or absence, in addition to calculating the percentages with which they occurred. It is noteworthy that these are supply reservoirs and therefore they may incur fluctuations in the water levels, although the greatest fluctuations are caused by water stress due to the long drought period which is typical in the semi-arid region [43, 53].

### 2.4 Perception of the local community

#### 2.4.1 Analysis of the perception

The perception of the interviewees was analysed using four basic questions related to the conservation (environmental quality) of reservoirs: 1 –Do you think the reservoir is conserved when you analyse the surrounding nature and water? 2 –Why do you think the reservoirs are conserved or not, considering the surrounding nature and water? 3 –What do you do to protect (conserve) the reservoir? and 4 –How would you classify the condition of the reservoir considering its surrounding nature and water?

At the time of the interviews, the interviewees were asked to consider the water quality of the reservoirs, the conditions of the surrounding vegetation, animals that live in the reservoir, and the “nature of the place” in order to formulate their answers. We hoped that they could provide answers which would refer to the conservation status of the reservoirs based on this analysis. Question 1 only admitted two possible answers (yes and no). The responses to question 2 were separated into two groups (the first group constituted an explanation for the reservoir not being conserved and the second group was an explanation for the reservoir being conserved), and the explanations were categorized depending on the similarity of the topic addressed in discourse [54]. The answers to question 3 were also categorized based on their similarity to the action taken to promote reservoir conservation. The following conservation levels were presented for question 4: 1 –Very good, 2 –Good, 3 –Average, 4 –Bad, and 5 –Terrible. The way in which the answers to these questions were analysed are shown in Table 1.

**Table 1:** Analysis of the responses provided by individuals from local communities.

Question	Analysis
1 –Do you think the reservoir is conserved when you analyse the surrounding nature and water?	Objective response with two options, yes or no. For the quantitative analysis, the positive responses to question 1 received a value of 1 and negative responses received a value of 0. The quantification of positive and negative responses was also performed to calculate percentages.
2 –Why do you think the reservoirs conserved or not, considering the surrounding nature and water?	Open question. Content analysis, considering the themes of the responses [54].
	Categories formed for the responses of people who did not consider the reservoir conserved–Reduction in water volume (RWV), Need to clean the reservoir (NCR), General pollution (GP), Pollution caused by sewage from the city (PSC), Use of pesticides (UP), Silting of reservoirs (SR), Bad smell (BS), Bad management (BM), Planting grass in the reservoir (PG), Production reduction (PR), Fishermen throw fish offal into the water (FTW), Reservoir covered by vegetation (RCV), Dead animals (DA), Houses with no septic tank (HST), Dead fish (DF), Unsafe drinking water (UW), Reservoir needs improvement (NIR), Deforestation (D), Water quality complaints (WQC), Predatory fishing (PF), Contaminated water has caused leptospirosis (CWL), Reduction of fish population (RFP).
	Category formed for the responses of people who consider the reservoir to be conserved–Only water option available (OWA), No pollution in the reservoir (NP), Not brackish water (NBW), Good quality (GQ), Water permits people to live in the region (WPR), Reservoir is good (RG), Low anthropogenic influence (LAI), Existence of good and bad places in the reservoirs (GBR), Permanence of riparian forest (PRF).
3 –What do you do to protect (conserve) the reservoir?	Open question. Content analysis, considering the themes of the responses [54].
	Conservation strategies performed by respondents—Avoid bathing in the reservoir (ABR), Avoid deforestation (AD), Avoid washing clothes in the reservoir (AWR), Avoid treating fish in the reservoir (ATR), Bury dead animals (BDA), Bury waste (BRW), Build septic tanks (BST), Burning of waste (BW), Feeding animals with macrophytes (FWM), Throwing waste into holes (JLG), Do not allow pesticide to flow into the reservoir (NAPR), Avoid planting grass (NPC), Do not pollute the water (NPW), Do not use pesticides (NUP), Do not allow sewage to enter the reservoir (NSER), Not throwing waste into the reservoir (NWR), Prevent animals from entering the water (PAEW), Remove vegetation from the reservoir (RVR), Use pesticide property (UPP), Save water (SW), Waste collection (WAC), Washing fishing traps away from the reservoir (WFTR).
4 –How would you classify the condition of the reservoir considering its surrounding nature and water?	Objective response with previously defined alternatives. Respondents chose one of the options for classifying the conservation status of the reservoir. Each option corresponded to a score (The score was not informed at the time of the interview): 1 –Very good, 2 –Good, 3 –Average, 4 –Bad and 5 –Terrible. For the purpose of calculating the Community Conservation Perception Index (CCPI) (See topic 2.4.2), points were assigned to each alternative so that the higher values expressed a higher level of reservoir degradation, and consequently less conservation.

We chose to consider higher scores for the answers to question 4 which expressed the worst ecosystem degradation level. This was done so that the answer scores could be used to calculate the Community Conservation Perception Index (CCPI), which is based on the Threat Criticality Index (See item 2.4.2), that uses increasing scores to express the intensity of ecosystem damage [62], and also so that the CCPI values could be interpreted similar to the Trophic State Index values (see item 2.2.1).

#### 2.4.2 Development of the Community Conservation Perception Index (CCPI)

The conception of the Community Conservation Perception Index (CCPI) came from the Threat Criticality Index developed by the World Wide Fund for Nature [62]. This index was also applied (with a different approach) to analyse the conservation threats to the reservoirs from the point of view of the local residents [52]. The Threat Criticality Index originally proposed by the WWF analyses the extension, severity and permanence of a threat in a determined area; each evaluated item receives a score (between 0 and 4) which expresses the damage intensity in an increasing manner. The values obtained for each item are multiplied, and the results indicate the impact level. The Criticality Index value for each threat is obtained by a ratio (division) of the threat level value by the maximum threat level value, considering the hypothesis that the three factors analysed (extension, severity, permanence) each present a maximum score (4). Thus, the index provides values between 0 and 1, with values closest to 1 indicating a higher threat criticality level.

The Community Conservation Perception Index (CCPI) was developed only based on question 4 (How would you classify the condition of the reservoir considering its surrounding nature and water? 1 –Very good, 2 –Good, 3 –Average, 4 –Bad, and 5 –Terrible), with the intention that the responses to the question would reflect a more general analysis of interviewee conservation perception of the reservoir. Each interviewee opted for one of the options which had scores varying between 1 and 5, with the greatest score reflecting the worst reservoir conservation. Different from the Criticality Index proposed by the WWF, it was not necessary to evaluate aspects such as extension, severity and permanence of specific threats to conservation, since the CCPI aimed to capture a more holistic expression of interviewee perception about the environment.

The scores given by all the individuals of the local community of a specific reservoir were added together for the final CCPI calculation, providing a value which was divided by the summation of the worst expected conservation state (assuming the possibility that all the interviewees chose the score of 5). The decision to add the scores of the recorded interviewee perceptions also differed for calculating the Criticality Index proposed by the WWF; however, this facilitated the calculations since smaller values were obtained by adding the scores, which would not be possible by multiplying the perception scores given by all the individuals in a community.

Thus, the final CCPI was calculated for each of the communities (Poções, Sumé, Traíras and Sabugí) using the following formula:

$$\mathrm{CCPI}=\Sigma_{Ptreal}/\Sigma_{Ptmax}$$

In which: the CCPI value corresponds to the Community Conservation Perception Index (with a value varying between 0 and 1), values close to 0 indicate a perception of good conservation, while values closer to 1 indicate worse conservation (greater perception of impacts on the ecosystem). *Σ*_*Ptreal*_ corresponds to the summation of the real score given by the whole community (the summation of the corresponding number of the response of each interviewee in the community). *Σ*_*Ptmax*_ corresponds to the summation of the total maximum expected scores for the community if all the interviewees opted for the highest score (5) (assuming a hypothetical situation where all the interviewees would classify the conservation of the reservoir as terrible).

The value obtained provided a summary of the conservation perception of the reservoir for each determined local community. The development of this index based on the perception of the interviewees facilitated performing a correlation analyses of the perception of communities with environmental metrics, such as the Trophic State Index. Although simple, the CCPI can represent one more metric for understanding the perception of local communities about the conservation of ecosystems.

### 2.5 Ethics declaration

The aims of this study were explained to the participants before each interview. Permission to register information was obtained through their signature on an Informed Consent Form (ICF), following the instructions of Resolution 466/2012 of the Brazilian National Health Council. Study approval was obtained through the Ethics Committee of the State University of Paraíba–UEPB (Licence No. 1.030.872).

### 2.6 Data analysis

#### 2.6.1 Statistical analysis of the data

The data referring to the interviewees’ perception about the conservation level of reservoirs (1 –Very good, 2 –Good, 3 –Average, 4 –Bad, and 5 –Terrible), Trophic State Index and anthropogenic influences in the riparian zone were processed with the Euclidean Distance coefficient. The spreadsheet of the taxonomic composition of macroinvertebrates was processed with the Bray-Curtis similarity index. The Euclidean Distance is the proper analysis for physical and chemical data or data of a non-biological nature, and represents the dissimilarity between the samples; Bray-Curtis is used for biological data, in which samples with many or without organisms may occur, and represents the similarity between the samples [63].

#### 2.6.2 Environmental quality of the reservoirs

The Trophic State Index (TSI), diversity, macroinvertebrate richness and human influences in the riparian zone were considered to classify the environmental quality of the reservoirs. Mean values and standard deviation were calculated for each reservoir for the Trophic State Index. The most abundant taxa were considered through their percentages to present data referring to benthic macroinvertebrates. The Shannon-Wiener diversity index [64] was calculated for each sampling location, the mean values of diversity per reservoir and the standard deviation were presented. The amount of different taxa in each reservoir was considered for richness. The percentages of inhabitation presence, electric transmission network, fences, pasture and agricultural zones were subsequently calculated for the data of anthropogenic influences in the riparian zone. The environmental (Trophic State Index and human influence in the riparian zone) and biological (Macroinvertebrate richness and diversity) data were compiled into a single set (grouping of data from all sampling months), thus, no analyses were performed by periods given that all sampling months were inserted in an extended dry period [53].

Significance analyses were performed to assess differences in the environmental quality of reservoirs. A Permutational Analysis of Variance (PERMANOVA) [63, 65] with 9999 permutations and α ≤ 0.05 and a Post-hoc test were carried out to evaluate the differences in the trophic state levels, anthropogenic influences in the riparian zone, and differences in the taxonomic composition (presence and absence of taxa at the sample site) of benthic macroinvertebrates between the reservoirs (the reservoir factor with four levels was considered for the analysis–Poções, Sabugí, Sumé and Traíras). Similarity Percentage Analysis (SIMPER) was used to evaluate the taxa that contributed up to 95% of the benthic macroinvertebrate. The Shannon-Wiener diversity index [64] was calculated with log_10_ for each sample site, using the abundance matrix to posteriorly compare the benthic macroinvertebrate diversity between the reservoirs. All statistical analyses were performed using the PRIMER-6 + PERMANOVA programme (Systat Software, Cranes Software International Ltd.) [63].

#### 2.6.3 Perception of the local community on the conservation of reservoirs and correlation of CCPI with the Trophic State Index, diversity, richness and anthropogenic influence in the riparian zone

First, the percentages of responses from individuals who considered the reservoir conserved and those who considered the reservoir not conserved were calculated for the responses to the question “Do you think the reservoir is conserved when you analyse the surrounding nature and water? Next, the percentage of responses that justified the conservation of the reservoir and of the responses that justified the non-conservation of the reservoir were also calculated for the responses to the question “Why do you think the reservoirs are conserved or not conserved, considering the surrounding nature and water?”.

Then, the percentage of responses was calculated for each conservation level considered by the interviewees for the question “How would you classify the condition of the reservoir considering the surrounding nature and water? (1 –Very good, 2 –Good, 3 –Average, 4 –Bad, and 5 –Terrible). A Permutational Multivariate of Variance (PERMANOVA, univariate) analysis with 9999 permutations and α ≤ 0.05 was subsequently used to ascertain the differences between the interviewee reservoir conservation perception levels from each community [63, 65], considering the spreadsheet with the different levels of categorised perception (1, 2, 3, 4, 5). We considered one factor (Reservoir) and four corresponding levels for each community local (Sumé, Poções, Traíras and Sabugí). This statistical analysis was also performed using the PRIMER-6 + PERMANOVA programme (Systat Software, Cranes Software International Ltd.) [63].

Next, a Pearson’s correlation analysis was used to analyse the correlation of the perception of reservoir conservation by the Community Conservation Perception Index (CCPI) and indicators. The CCPI was correlated with the mean values of the Trophic State Index, mean values of diversity, total species richness and percentages of anthropogenic influences in the riparian zone of each reservoir (Residences, agriculture, transmission lines, fences and pasture). The R statistical environment software programme (The R Development Core Team) [66] was used for this analysis.

#### 2.6.4 Relation of the environmental quality of reservoirs with the number of conservation strategies performed by the local community

The different types of answers to the question “What do you do to protect (conserve) the reservoir?” were quantified to express reservoir conservation strategies in each local community. After defining the environmental quality of the reservoirs (Procedure described in topic 2.6.2), a table was drawn up listing the conservation strategies adopted by individuals from each local community. Then, the percentages of citations of each strategy in the local communities were calculated as a way to represent the proportions of the types of strategies.

## 3. Results

### 3.1 Environmental quality of the reservoirs

There were significant differences for the Trophic State Index between the reservoirs, except when considering Poções x Traíras and Sumé x Sabugí (S1 Table). The average trophic state in Poções was 68 (standard deviation of ± 8.2), in Sumé 54 (standard deviation of ± 9.6), in Traíras 71 (standard deviation of ± 10.3) and Sabugí 53 (standard deviation of ± 9.4). Considering the average values for the Trophic State Index, Poções and Traíras were classified as eutrophic and Sumé and Sabugí as mesotrophic.

There was a difference in taxonomic composition between the reservoirs when analysing the macroinvertebrates, with the exception of the Poções x Traíras and Sabugí x Sumé reservoirs (S2 Table). Oligochaetes and *Melanoides tuberculata* (Müller, 1774) were found in the communities of all the reservoirs, whereas *Tanytarsas*, *Aesheum*, *Fissimentum* taxa only occurred in Sabugí, with greater Chironomidae richness in this reservoir (Table 2). The *Corbicula largillierti* (Philippi, 19844) mollusc was only found in Sumé (Table 2). The greatest diversities occurred in the Sabugí and Sumé reservoirs, with a mean of 0.53 (standard deviation of ± 0.18) and mean of 0.29 (standard deviation of ± 0.15), respectively. The lowest diversities occurred in the Traíras and Poções reservoirs, with a mean of 0.24 (standard deviation of ± 0.19) and 0.21 (standard deviation of± 0.13), respectively. Higher richness values were registered in the reservoirs in Sumé (36) and Sabugí (32), and lower in Poções (21) and Traíras (16).

**Table 2:** Contribution of up to 95% of the benthic macroinvertebrate in the study reservoirs.

	Poções	Sumé	Traíras	Sabugí
**Mollusca**				
*Melanoides tuberculata*	42.81	55.13	25.61	7.25
*Corbicula largillierti*		0.40		
**Annelida**				
Oligochaeta	53.72	38.54	66.60	41.93
**Diptera**				
Ceratopogonidae				0.42
**Chironomidae**				
*Aedocrytus*			5	4.03
*Aesheum*				5.72
*Coelotanypus*				4.10
*Fissimentum*				1.98
*Goeldichironomus*				11.92
*Polypedilum*				4.11
*Tanytarsus*				13.22

Percentage (%) values obtained through the SIMPER analysis.

The landscape of the riparian zone was different between the Poções x Traíras and Sabugí and Sumé reservoirs (S3 Table). The Sabugí reservoir presented low development percentages of agricultural activities (7%); on the other hand, the Poções and Traíras reservoirs together presented the highest proportions of agricultural activity development (19% and 24%, respectively) and presence of residences (22% and 24%, respectively) (Table 3).

**Table 3:** Mean values (TSI, DIV), absolute values (RICH), and percentages (RESID, AGRIC, LINT, FEN, PAS) of the variables used to define the environmental quality of the reservoirs.

Reservoirs	TSI	DIV	RICH.	RESID %	AGRIC %	LINT %	FEN %	PAS%
**Sumé**	54	0.53	36	19	20	17	24	19
**Sabugí**	53	0.29	32	22	7	30	22	18
**Poções**	68	0.24	21	22	19	17	23	19
**Traíras**	71	0.19	16	24	24	17	25	20

TSI (Trophic State Index), DIV (mean diversity by reservoir), RICH (total species richness by reservoir), RESID (percentage of households), AGRIC (percentage of agriculture), LINT (percentage of transmission lines), FEN (percentage of fences), PAS (percentage of pasture).

Considering the values and differences related to the Trophic Status Index, diversity and richness of the benthic macroinvertebrate community and anthropogenic influence in the riparian zone (Table 3), the Poções and Traíras reservoirs were confirmed to have the worst environmental quality (Worst conservation status), and the Sumé and Sabugí reservoirs were confirmed as having better environmental quality in relation to the others (Better conservation status).

### 3.2 Perception of the local community on reservoir conservation and correlation of the Community Conservation Perception with the Trophic Status Index, diversity, richness and conservation of the riparian zone

According to the question “Do you think the reservoir is conserved when you analyse the surrounding nature and water?”, the majority of interviewees (67.5%, n = 85) affirmed that the reservoirs were not conserved, with the exception of residents in the area surrounding the Sabugí reservoir, whose majority affirmed that the reservoir was conserved (60%, n = 21) (Table 4).

**Table 4:** Perception of the interviewees in terms of the conservation of the reservoir where they reside, hydrographic basins of the Paraíba and Piranhas-Assú Rivers, Brazil.

	Preserved	Not Preserved
Poções	10.50% (n = 4)	89.50% (n = 34)
Sumé	36.40% (n = 8)	63.60% (n = 14)
Traíras	26.70% (n = 8)	73.30% (n = 22)
Sabugí	60.00% (n = 21)	40.00% (n = 14)

**Fig 2:**
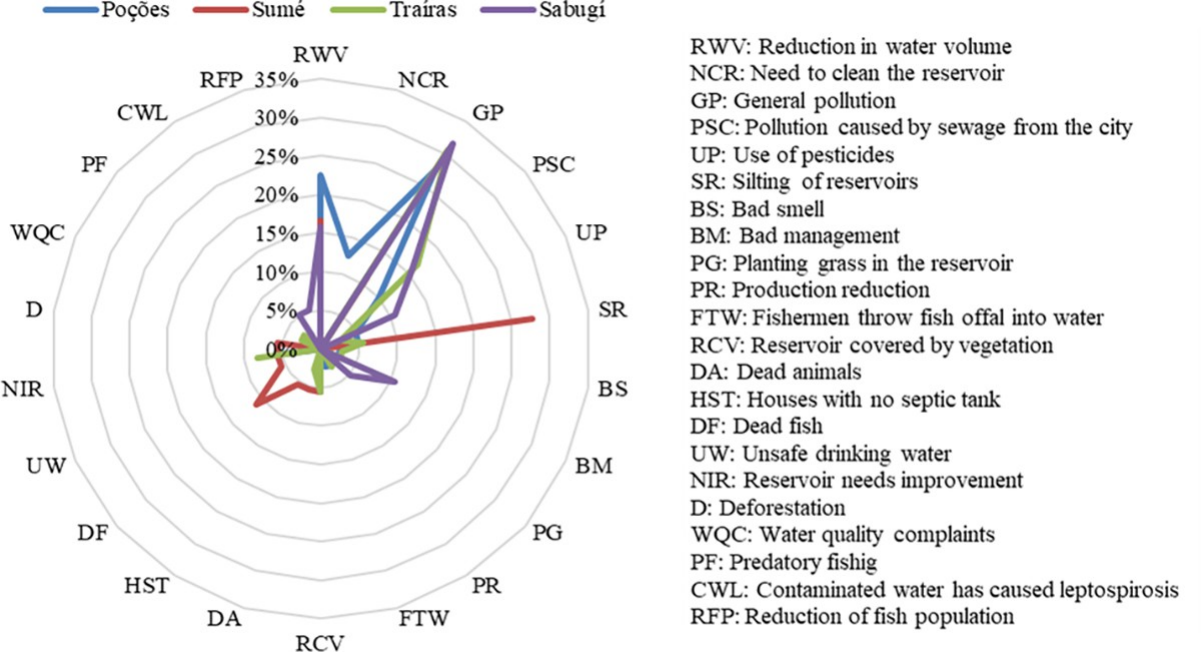
Justifications for the lack of conservation of the reservoirs. The main justifications were reduction in water volume (RWV), general pollution (GP) and silting of reservoirs (SR).

Considering the question “Why do you think the reservoirs are conserved or not conserved, considering the surrounding nature and water?”, the justifications for the poor conservation of the reservoirs which stood out the most were the reduction in water volume (RWV), general pollution (GP) and silting of reservoirs (SR) (Fig 2). Some answers reflected the community perceptions on changes in water volume and pollution in reservoirs:

*“The reservoir is bad because it is dry”* (Interviewee from the community around the Poções reservoir).

*“…it’s bad because the water level is low*” (Interviewee from the community around the Sumé reservoir).

*“It is dirty*, *pollution from the cities*, *like the old soap factory…”* (Interviewee from the community around the Traíras reservoir).

**Fig 3:**
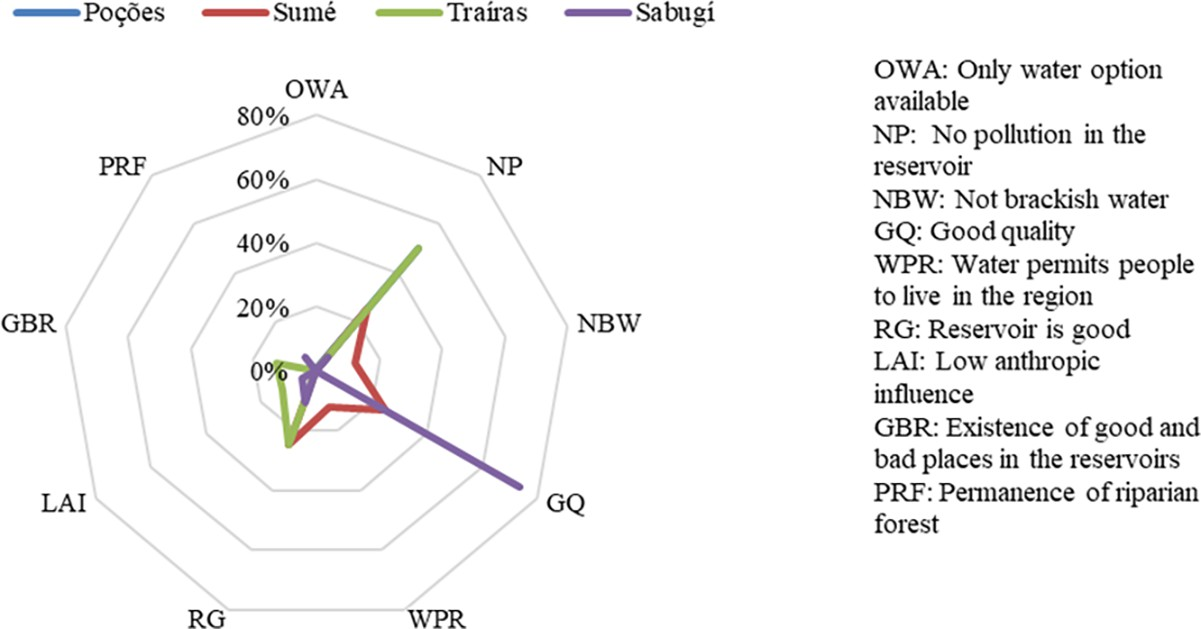
Interviewee justifications for considering the reservoirs to be conserved. The main justifications were water to be of good quality (GQ) and no pollution in the reservoir (NP).

The justifications which stood out among the interviewees who considered the reservoirs to be conserved were those that considered the water to be of good quality (GQ), and the fact that they considered that there was no pollution in the reservoir (NP) (Fig 3). The comments below present the justifications given by the interviewees who considered the reservoirs to be conserved:

*“It is the best in the region*” (Interviewee from the community around the Sabugí reservoir, referring to the quality of the water).

*“If it was full it would be better*, *it’s all we have*” (Interviewee from the community around the Sabugí reservoir).

*It’s good because there is no better water”* (Interviewee from the community around the Poções reservoir).

In considering the question “How would you classify the condition of the reservoir, considering the surrounding nature and water?”, some of the interviewees (39.47%) of the community around the Poções reservoir classified the condition of the reservoir as bad, whereas 50% people interviewed in Sumé classified the conservation state as good or very good. The majority of interviewees from the community around the Traíras reservoir (64.51%) classified the conservation state as bad, and 51.42% interviewees from Sabugí classified the reservoir as being in a good state of conservation (Table 5). The Community Conservation Perception Index (CCPI) indicated that the Poções and Traíras reservoirs (0.69 and 0.66, respectively) had the worst conservation states, whereas the Sumé and Sabugí reservoirs (0.54 and 0.56, respectively) presented less degradation (Better conservation status) (Table 5). There was a difference between communities in the perception of the reservoir conservation level; from a statistical point of view, the first two communities (Poções and Traíras) presented different conservation perceptions in relation to the last two communities (Sumé and Sabugí) (S4 Table).

**Table 5:** Classification of data by the interviewees on the conservation of the reservoirs and the Community Conservation Perception Index (CCPI), hydrographic basins of the Paraíba and Piranhas-Assú Rivers, Brazil.

	Very good (1)	Good (2)	Average (3)	Bad (4)	Terrible (5)	CCPI *= Σ*_*Ptreal*_*/Σ*_*Ptmax*_
Sumé	9.10% (n = 2)	40.90% (n = 9)	18.20% (n = 4)	31.80 (n = 7)	*	0.54
Sabugí	*	51.42% (n = 18)	14.30% (n = 5)	34.30% (n = 12)	*	0.56
Traíras	*	32.30% (n = 10)	3.22% (n = 1)	64.50% (n = 20)	*	0.66
Poções	*	13.15% (n = 5)	36.84% (n = 14)	39.50% (n = 15)	10.52% (n = 4)	0.69

*Represents categories that were cited by the interviewees.

**Fig 4:**
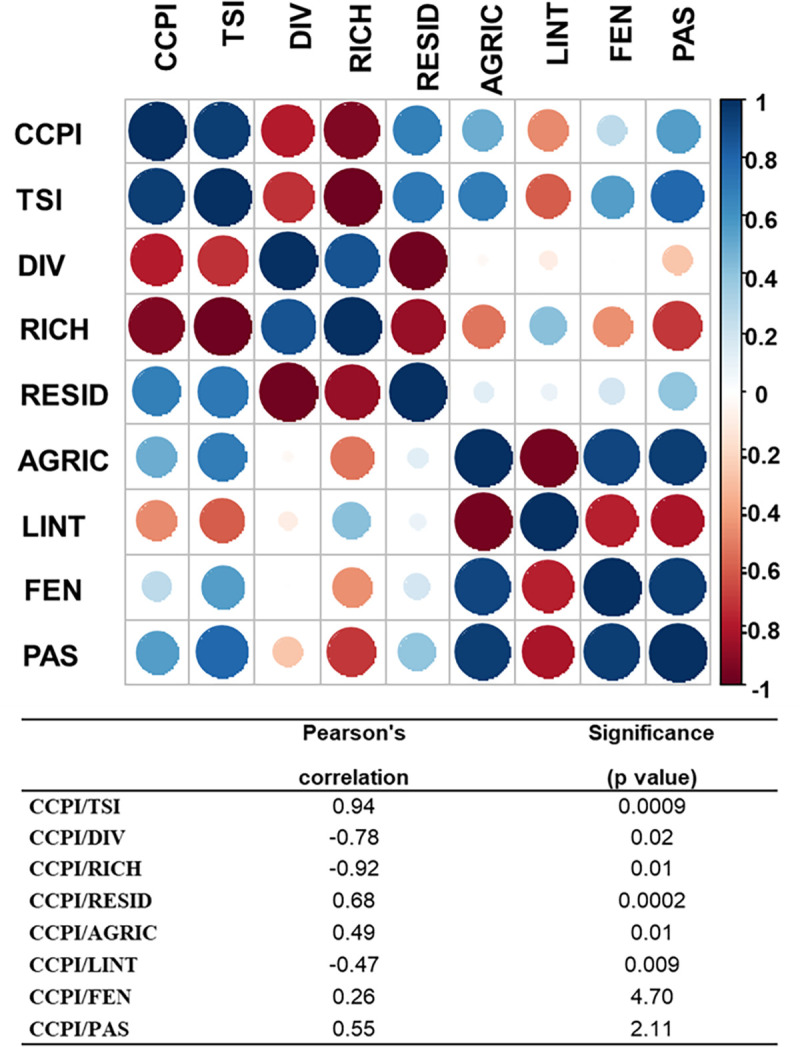
Graph representing the Pearson’s correlation of Community Conservation Perception Index with Trophic State Index, average diversity, total species richness, and anthropogenic influences in the riparian zone. The table presents the correlation values and the p-value for each performed correlation CCPI (Community Conservation Perception Index), TSI (Trophic State Index), DIV (average diversity by reservoir), RICH (total species richness by reservoir), RESID (percentage of residences), AGRIC (percentage of agriculture), LINT (percentage of transmission lines), FEN (percentage of fences), PAS (percentage of pasture). Circles in shades of blue indicating positive correlations, circles in shades of red indicating negative correlations.

The results demonstrate that the CCPI presents a positive and significant correlation with the Trophic State Index (Pearson’s r = 0.94; p = 0.0009), a negative correlation with diversity (Pearson’s r = - 0.78; p = 0.02) and species richness (Pearson’s r = - 0.92; p = 0.01). Considering the anthropogenic influences on the riparian zone of the reservoirs, the CCPI showed a positive and significant correlation with the presence of residences (Pearson’s r = 0.68; p = 0.0002) and development of agricultural activities (Pearson’s r = 0.49; p = 0.01). A positive but non-significant correlation was found between CCPI and fences (Pearson’s r = 0.26; p = 4.7) and pastures (Pearson’s r = 0.55; p = 2.11). There was also a negative and significant correlation between CCPI and the presence of transmission lines (Pearson’s r = -0.47; p = 0.009). The graph and results of the correlations can be seen in Fig 4.

### 3.3 Relation of the environmental quality of reservoirs with the number of conservation strategies performed by the local community

Taking into consideration the question “What do you do to protect (conserve) the reservoir?”, the measures applied by the interviewees for the conservation of the reservoir which stood out the most were: burning of waste (BW) and the act of not throwing waste into the reservoir or in its proximity (NWR) (Table 6).

**Table 6:** Presentation of conservation strategies carried out by each local community to maintain reservoir conservation.

Environmental quality of the reservoir	Reservoir and local community	Actions performed by the local community that promote reservoir conservation
**In reservoirs with worse environmental quality—Worst conservation state**	Poções	30% Burning of waste (BW)
		24% Not throwing waste into the reservoir (NWR)
		14% Do not allow sewage to enter the reservoir (NSER)
		8% Prevent animals from entering the water (PAEW)
		6% Avoid washing clothes in the reservoir (AWR)
		4% Build septic tanks (BST)
		2% Avoid planting grass (APG)
		2% Avoid throwing fish residues in the reservoir (ATR)
		2% Bury dead animals (BDA)
		2% Do not allow pesticide to flow into the reservoir (NAPR)
		2% Do not pollute the water (NPW)
		2% Do not use pesticides (NUP)
		2% Waste collection (WAC)
	Traíras	59.18% Burning of waste (BW)
		10.20% Build septic tanks (BST)
		8.16% Waste collection (WAC)
		6.12% Do not allow sewage to enter the reservoir (NSER)
		6.12% Not throwing waste into the reservoir (NWR)
		2.04% Avoid deforestation (AD)
		2.04% Avoid washing clothes in the reservoir (AWR)
		2.04% Bury dead animals (BDA)
		2.04% Use pesticides properly (UPP)
		2.04% Save water (SW)
**In reservoirs with better environmental quality—Better conservation status**	Sumé	48.94% Burning of waste (BW)
		10.64% Build septic tanks (BST)
		8.51% Not throwing waste into the reservoir (NWR)
		8.51% Waste collection (WAC)
		4.26% Avoid deforestation (AD)
		4.26% Do not use pesticides (NUP)
		2.13% Avoid bathing in the reservoir (ABR)
		2.13% Avoid throwing fish residue in the reservoir (ATR)
		2.13% Bury waste (BRW)
		2.13% Do not allow sewage to enter the reservoir (NSER)
		2.13% Remove vegetation from the reservoir (RVR)
		2.13% Throwing waste into holes (TWH)
		2.13% Washing fishing traps away from the reservoir (WFTR)
	Sabugí	55.17% Burning of waste (BW)
		13.79% Do not use pesticides (NUP)
		10.34% Build septic tanks (BST)
		10.34% Remove vegetation from the reservoir (RVR)
		3.45% Feeding animals with macrophytes (FWM)
		3.45% Do not allow sewage to enter the reservoir (NSER)
		3.45% Not throwing waste in to the reservoir (NWR)

The local communities of Poções and Sumé each cited 13 strategies to promote reservoir conservation, while the communities of Traíras and Sabugí cited 10 and 7 strategies, respectively. The strategies with the highest citation percentages for the Poções community were “burning of waste” (30%) and “not throwing waste into the reservoir” (24%); for Sumé it was “burning of waste” (48.94%) and “build septic tanks” (10,64%); for Traíras “burning of waste” (59.18%) and “build septic tanks” (10.20%); and for Sabugí it was “burning of waste” (55.17%) and “do not use pesticides” (13.79%) (Table 6).

## 4. Discussion

### 4.1 Environmental quality of the reservoirs (conservation state)

The environmental quality of the reservoirs was indicated by the Trophic State Index, species richness and diversity, and anthropogenic influences in the riparian zone, as well as by the differences in these variables between the reservoirs. The artificial eutrophication process caused by point or diffuse sources of organic pollution [51, 67], or even by the reduced water volume of reservoirs in dry periods, which causes a concentration of nutrients in the water due to evaporation (a common event in reservoirs of Brazilian semi-arid region) [50], affects the establishment of species which are sensitive to pollution [24] and the water use of reservoirs by local communities [43].

The differences between benthic macroinvertebrate taxa and diversity, also highlighting the environmental quality of the study ecosystems, corroborate the trophic state of the reservoirs, and anthropogenic influences in the riparian zone. The low diversity and richness values in these eutrophic reservoirs reflect homogenization of the benthic macroinvertebrate between the reservoirs. Hill and Wood [68] found less dissimilarity in benthic composition between anthropogenised lakes. Taxonomic composition reduced with the increase in anthropogenic pressure, resulting in the prevalence of generalist species in more impacted areas [69–71]. Thus, reductions in diversity can be related to greater environmental degradation in reservoirs with greater levels of anthropogenic influences in the riparian zone and higher Trophic State Index values [72, 73].

It was found that increases in agricultural areas and decreases in riparian vegetation are the main factors regarding anthropogenic influences which influence Chironomidae (Diptera) communities in the riparian zone [74]. Previous studies have also shown similar results [69, 75, 76]. The presence of residences is also an indication of the pressure level to which the reservoirs are subjected; homes in rural areas do not usually have a basic sanitation system, which contributes to intensify eutrophication of aquatic ecosystems [77]. Moreover, alterations in the riparian zone may cause changes in runoff, silting, soil erosion and vegetation loss [38, 78].

The indicators analysed, especially the Trophic Status Index, show the impacted condition of the reservoirs mainly due to the extreme drought period in which this study was carried out, which negatively impacted the environmental quality of reservoirs (mainly Traíras and Poções) [43]. Our records occurred in a period considered as being the worst drought in the last 50 years (2011–2016), which made it impossible for these environments to reach their maximum ecological potential [7, 47]. However, within the impact gradient, it was possible to differentiate reservoirs with fewer impacts (Sumé and Sabugí) from more impacted reservoirs (Poções and Traíras).

### 4.2 Perception of the local community on reservoir conservation and correlation of the Community Conservation Perception with the Trophic Status Index, diversity, richness and conservation of the riparian zone

When comparing the environmental quality of the reservoirs, it can be seen that the perception of the local community corresponds to their conservation status, and this data is corroborated by the answers given by individuals from the communities. Local communities also presented feasible answers to justify the poor conservation of reservoirs. The existence of the relationship of local community perception and the trophic state of lakes has been previously registered in a study by Kooyoomijan and Clesceri [79]. In this study, the authors reported less complaints about water quality by communities using water from oligotrophic lakes than in communities using water from eutrophic lakes.

In the same communities and reservoirs analysed herein, Azevêdo et al. [43] indicated that local community notice alterations in water quality based on interactions developed in the ecosystem. As they often observe the reservoir, they carry out activities which depend on the perception of change in the volume and water quality (such as fishing, agricultural, and domestic water uses), often changing the ways of water used in response to the loss of their water quality. It should be considered that we analysed the perception of the local communities at a time of extreme drought [53], and thus it is necessary to consider that perceptions change as reservoirs improve their environmental quality with the increase in water volume.

The correlations between the CCPI and the Trophic State Index, species richness and diversity, and the presence of agricultural activities and residences in the riparian zone ratify that the perception of the local community (expressed by the CCPI) is correlated with the conservation status of the reservoirs during the study period. These relationships show that the perception of local communities must be considered in reservoir conservation, considering that they have the potential to express the conservation status of the ecosystem. Furthermore, it is necessary to go beyond the conservation of species and ecosystems [80, 81].

Governments and managers must enable and implement collaborative participation of local communities in the conservation and management processes [82]. Cummins et al. [83] showed a practical example of the possibilities of joint work between local community and scientists, with political support by the Apsaálooke (Crow) tribe in south-central Montana. A partnership was carried out in this situation to examine groundwater and surface water contaminants through community-based participatory research (CBPR). By implementing CBPR, it was possible to carry out participatory water quality monitoring, obtain health population data, and obtain funding to carry out projects in favour of the community and environment. Other studies have shown the importance of including and making communities responsible for participatory water management processes [i.e.: 84, 85]. Collaborative management for reservoirs could be enacted in different ways from including local communities in actions such as: 1—pollution investigation; 2—collection of parameters related to water quality; 3—access to scientific data collected, which should be shared in order to be understood by the community, 4—participation in the design of conservation strategies; and 5—encouraging the development of sustainable ecological tourism with the participation of local guides.

It was possible to summarize the perception of the local community in a single quantitative data through the CCPI, which can facilitate defining favourable conservation strategies to be analysed and monitored based on quantitative information. However, it should be considered that qualitative information is essential to broaden understanding of the environmental quality of the reservoir, since this information offers complementary and different answers on the environmental quality between reservoirs, as observed in this study when analysing the justifications for the good and bad conservation of the reservoir.

One of the greater challenges in the Brazilian context is to guarantee the effective participation of the local communities in the Hydrographic Basin Committees, considering that there is little understanding of the committee staff and their importance, in addition to the impossibility of participation by a greater number of water users. In this sense, legislation such as Law 9433 [32] and Resolution No. 5 de 2000 [33] could be changed/amended to include the CCPI analysis and quantitative information on the perceptions of local communities about environmental uses and problems in the basins, guaranteeing broader participation of water users in the Committees. For example, the CCPI can be periodically applied in implementing mitigating measures to assess whether the local community has noticed an improvement in the conservation status of ecosystems, since the perceptions and results provided by the CCPI may vary over time, and considering that the perceptions of the local communities may vary as a function of the environmental conditions prevailing at the CCPI application time. This is important information for managers who need to get feedback on the implemented measures. However, it must be clarified that the use of CCPI does not rule out an analysis of qualitative data, which is essential for identifying problems and specific demands of each local community.

### 4.3 Relation of the environmental quality of reservoirs with the number of conservation strategies performed by the local community

Unlike what was expected, the greatest number of strategies developed by local communities to promote reservoir conservation did not occur in communities where the reservoirs were classified as having higher environmental quality (better conservation status). Garbage burning was one of the strategies that was highlighted, and it is justified because there is no collection carried out by public agencies in rural areas, with few exceptions [86, 87]. Although it promotes carbon dioxide emission, it seems to be less harmful than leaving the waste in the environment.

The strategies in locations where the reservoirs had the worst quality (worst conservation state) may be inefficient due to the pollution pressure and water stress to which the reservoirs were submitted during the research period [50, 51, 67]. A study carried out by Azevêdo et al. [52] in the same communities and reservoirs shows that local communities indicate the discharge of waste, mainly from urban areas, as the main factor which threatens the conservation of reservoirs, as well as sewage input from cities upstream of the reservoir. Another important factor which contributed to the worse environmental quality of the reservoirs was the prolonged drought that occurred from 2011 to 2016 [53], a period that encompasses the years 2014 and 2015 (when this study was carried out). As discussed earlier (see topic 4.1), drought promotes high water evaporation in the reservoir, causing an increase in nutrient concentration and consequent eutrophication [50].

Implementing more strategies in reservoirs with lower environmental quality (worse conservation state) may have occurred as a result of people in these locations starting to adopt different types of remedial and non-preventive strategies to mitigate the poor quality of the reservoir. This comes from the perspective that human beings could develop different strategies to cope with environmental changes in the ecosystems, whether they are natural or caused by humans [88, 89]. However, it is necessary to recognize that poorer communities, like many local communities, do not have the technological and financial resources to deal with these changes, which evidences the need for participatory performance of the government.

Azevêdo et al. [90] reported that local communities in the reservoirs studied herein use local indicators to assess water quality, with colour and water smell being the most dominant, although these indicators cannot solely define its quality. These communities have also been found to change water use as water quality declines due to prolonged drought, which increases nutrient concentrations and causes algal blooms in reservoirs. As the water loses its quality, the community stops using it for more noble purposes such as drinking and cooking food [43].

However, it is necessary to find out whether conservation strategies are routinely implemented in the reservoirs, regardless of their environmental quality, or whether communities start to increase the quantity and different types of strategies only when they notice the environmental quality of the reservoirs deteriorating.

## 5. Conclusion

The perception of the local community about the conservation status of the reservoirs is related to the trophic status, diversity, macroinvertebrate species richness, the presence of residences and agriculture in the riparian zone. However, the number of conservation strategies adopted by local communities did not reflect the conservation status of the reservoirs. The data collected in this study can serve as a basis to develop reservoir conservation actions, considering the particularities of local communities and ecosystems.

Thus, valuing the participation of residents that live around hydrographic basins is of fundamental importance for management and participative conservation, since it can enable public participation in management and environmental conservation and trigger the early resolution of conflicts between interested parties in society.

## Supporting information

S1 File (PDF)

S1 Data (XLSX)

S2 Data (XLSX)

S3 Data (XLSX)

S4 Data (XLSX)

S5 Data (XLSX)

S6 Data (XLS)

S1 Table Results of the PERMANOVA analysis and Post-hoc tests for the evaluation of the Trophic State Index of the study reservoirs, hydrographic basins of the Paraíba and Piranhas-Assú Rivers, Brazil. X corresponds to the test ratio between one reservoir and another.
(DOCX)

S2 Table Results of the PERMANOVA and Post-hoc tests with dissimilarity of the taxonomic benthic macroinvertebrate composition for the study reservoirs, hydrographic basins of the Paraíba and Piranhas-Assú Rivers, Brazil. X corresponds to the test ratio between one reservoir and another.
(DOCX)

S3 Table Results of the PERMANOVA analysis and Post-hoc tests for the anthropogenic influences in the riparian zone, hydrographic basins of the Paraíba and Piranhas-Assú Rivers, Brazil. X corresponds to the test ratio between one reservoir and another.
(DOCX)

S4 Table Results of the PERMANOVA analysis and Post-hoc tests for the evaluation of the different conservation perceptions between the study reservoir communities, hydrographic basins of the Paraíba and Piranhas-Assú Rivers, Brazil. X corresponds to the test ratio between one local community and another.
(DOCX)
