# Supplementary material for: Perception of the local community: What is their relationship with environmental quality indicators of reservoirs?
Source: PLoS One. 2022 Jan 21;17(1):e0261945. doi: 10.1371/journal.pone.0261945 (PMC8782485; doi:10.1371/journal.pone.0261945)
Supplement: S1 File — (PDF) [file pone.0261945.s001.pdf]

Questions used for the manuscript (Perception of the local community: what is their relationship with environmental quality indicators of reservoirs?) are marked in yellow.

FORM

City/Rural area:_____________Date:________

Name:__________________________________

_________________Age:_______Sex:______

Main occupation: __________________

Others: ________________________________

Marital status: Married ( ) Not married ( ) Divorced ( )

Have children? Yes ( ) Not ( )

How many children?_____________________

Education level:

You don't read and you don't write ( )

You can write only your name ( )

you read and write ( )

Elementary school:

Complete ( ) Incomplete ( )

High school:

Complete ( ) Incomplete ( )

University:

Complete ( ) Incomplete ( )

Activity, Monthly Income and Social Security Data:

What is your monthly income? ____

What is your religion? ____________________

1- How long have you lived in this place?

2- What does the water in this reservoir mean to you?

3- Talk about the uses of water from this reservoir.

4- For what purpose do you use the water in the reservoir?

(1) Drink (2) Recreation (3) Bath
(4) Domestic use (4) Animal use (5) Other:
____

In case of domestic use.
Which?
____

In case of animal use. Which?
____

5- Can you see changes in the water quality of this reservoir?

(1) Yes (0) Not

6- If yes, what changes can you notice when the water is bad?

(1) Bad smell. Which?
(2) Presence of aquatic plants. Which?
(3) Presence of animals. Which?
(4) Change in water color. Which color?
(5) Disappearance of plants. Which?
(6)Disappearance of animals. Which?
(7) Change in taste of water. Which?
(8) Other:______________________________

7- If so, what changes can you notice when the water is good?
(1) Odor. Which?
(2) Presence of aquatic plants. Which?
(3) Presence of animals. Which?
(4) Water color. Which color?
(5) Disappearance of plants. Which?
(6) Disappearance of animals. Which?
(7) Change in taste of water. Which?
(8) Other:______________________________

8- Can you see changes in the water quality of the reservoir at a time of year?

9- When it rains, do you notice changes in water quality? If yes, which one?

10- Do you develop agricultural activity in this area?

(1) Yes (0) Not

11- At where?
(1) Next to the reservoir. How far?
(2)A little far. How far?
(3) Far. How far?

12- Do you raise animals (Eg.: Chickens, pigs, cows, goats)?
(1) Yes (0) Not

13- If yes, which ones?

14- At where?
(1) Next to the reservoir. How far?
(2) little far. How far?
(3) Far. How far?

15- Do you use any agricultural defensives? Which?

16- What human actions do you see that can reduce the quality of the reservoir?

17- Where does the water you drink come from?
(1) Cistern
(2) It comes from the reservoir near your house.
(3) It comes from another reservoir.
(4) Waterholes (Cacimba).
(5) Wells.
(6) River.
(7) Groundwater (Olho d'água).
(8) Other:_________________________

18- How does the water from the reservoir reach your home?
(1) Water is piped using pumps.
(2) Piped water without pump use.
(3) Human traction.
(4) Animal traction. Which?
(5) Other:_________________________

19- In your family or in known families, is there any occurrence of diseases related to the use of water from this reservoir?

(1) Yes (0) Not

20- What diseases?

21- What do you think is the cause of each disease?

22- At what time of year are these diseases more frequent?

23- Do you treat the water in the reservoir? If yes, how?
1(24)- Do you think the reservoir is conserved when you analyse the surrounding nature and water?

(1)Yes (0) Not

2(25)- Why do you think the reservoirs are conserved or not, considering the surrounding nature and water?

26- Do you adopt measures for the conservation (care) of the reservoir?
(1) Yes (0) Not

3(27)- What do you do to protect (conserve) the reservoir?

4(28)- How would you classify the condition of the reservoir considering its surrounding nature and water?

(1) Very good (2) Good,
(3) Average (4) Bad
(5) Terrible

29- How often do you go to the reservoir?

30- What do you do when you go to the reservoir?

31- How did you learn about changes in water quality?
1- With your father or mother.
2- Friends of the same generation.
3- From the experience in the reservoir.
4- With older people other than your parents.
5- Mídia (Eg.: Tv, radio)
6- With your teacher
7- Other________________________________

Comments:

Questões utilizadas para o manuscrito (Perception of the local community: what is their relationship with environmental quality indicators of reservoirs?) estão marcadas em amarelo.

FORMULÁRIO

Cidade/Sítio:________________Data:______

Nome:____________________________________

________________idade:_______Sexo:____

Atividade principal:

_______________Outras: _______________

Estado Civil: Casado ( ) Solteiro ( )

Separado ( )

Tem filhos? Sim ( ) Não ( )

Quantos?____

Grau de instrução:

Não lê e não escreve ( )

Apenas escreve o nome ( )

Ler e escreve ( )

Ensino Fundamental:

1° ciclo comple ( ) 1° ciclo incompleto( )

2° ciclo completo ( ) 2° ciclo incompleto ( )

Nível médio: Completo ( ) Incompleto ( )

Nível superior: Completo ( ) Incompleto( )

Dados da Atividade, Renda Mensal e Previdência Social

Qual a sua renda mensal? ____

Qual a religião?__________________

1- A quanto tempo você mora nesta localidade?

2- O que a água do açude representa para você?

3- Fale sobre os usos da água do açude.

4- Para qual finalidade você usa a água do reservatório?

(1) Beber (2) Recreação (3) Banho
(4) Uso doméstico (4) Uso animal (5) Outro

Em caso de uso doméstico.
Quais?________________________________
Em caso de uso animal.
Quais?______________________________

5- Você consegue perceber alterações na qualidade da água?
(1) Sim (0) não

6- Em caso afirmativo, quais são as alterações que você consegue perceber quando a água está ruim?

(1) Mal cheiro. Qual?
(2) Aparecimento de plantas aquáticas. Quais?
(3) aparecimento de animais. Quais?
(4) alteração da cor da água. Qual cor?
(5) Desaparecimento de plantas. Quais?
(6) Desaparecimento de animais. Quais?
(7) Alteração do gosto da água. Qual?
(8) Outros:______________________

7- Em caso afirmativo, quais são as alterações que você consegue perceber quando a água está boa?
(1) Cheiro. Qual?
(2)Aparecimento de plantas aquáticas. Quais?
(3) Aparecimento de animais. Quais?
(4) Cor da água. Qual cor?
(5) Desaparecimento de plantas. Quais?
(6) Desaparecimento de animais. Quais?
(7) Alteração do gosto da água. Qual?
(8) Outros:______________________

8- Você consegue perceber alteração da qualidade da água em algum período do ano?

9- Quando chove você percebe alguma alteração na qualidade da água? Se sim, quais?

10- Você desenvolve atividade agrícola?
(1) Sim (0) Não

11- Onde?
(1) Próximo do açude. Distância?
(2) Um pouco distante. Distância?
(3) Longe. Distância?

12- Você desenvolve alguma criação de animal?
(1) Sim (0) não

13- Se sim. Qual?

14- Onde?
(1) Próximo do açude. Distância?
(2) Um pouco distante. Distância?
(3) Longe. Distância?

15- Usa algum defensivo agrícola? Qual ou Quais?

16- Quais as ações humanas que você percebe que tem diminuído a qualidade do açude?

17- De onde vem a água que você utiliza para beber?

(1) Cisterna
(2) Do Açude próximo a sua casa.
(3) De outro açude.
(4) Cacimba.
(5) Poço.
(6) Rio.
(7) Olho d'água.
(8) Outo:_________________________

18- De que maneira a água do reservatório chega a sua casa?
(1) A água é encanada com uso de bombas.
(2) A água encanada sem bomba.
(3) Tração humana
(4) Tração animal. Qual?
(5) Outro:___________

19- Na sua família ou conhecidos existe ocorrência de doenças relacionadas ao uso da água deste reservatório?

(1) Sim (0) Não

20- Quais as doenças?

21- A que você atribui cada doença?

22- Em qual período do ano estas doenças são mais frequentes?

23- Você realiza algum procedimento para tratar a água do açude? Se sim, Qual (is)?

1(24)- Você acha que o reservatório é conservado quando você analisa a natureza e a água ao redor?

(1)Sim (0) Não

2(25)- Por que você acha que os reservatórios são conservados ou não, considerando a natureza do entorno e a água?

26- Você adota medidas para a conservação (cuidado) do açude?

(1) Sim (0) Não

3(27)- O que você faz para proteger (conservar) o reservatório?

4(28)- Como você classificaria o estado do reservatório considerando a natureza do entorno e as águas?

(1) Muito boa (2) Boa
(3) Intermediária (4) Ruim
(5) Terrível

29-Com que frequência você vai ao açude?

30-Quais atividades você desempenha no açude?

31-Como você obteve s informação par saber sobre a mudança da qualidade da água?

1-Pais 2- amigos da mesma geração
3-você mesmo 4-pessoas mais velhas, que não seus pais
5-Mídia (Tv, radio, etc)
6- professor
7- outro____

Observações:
