# Supplementary material for: Perception of the local community: What is their relationship with environmental quality indicators of reservoirs?
Source: PLoS One. 2022 Jan 21;17(1):e0261945. doi: 10.1371/journal.pone.0261945 (PMC8782485; doi:10.1371/journal.pone.0261945)
Supplement: S1 Table — X corresponds to the test ratio between one reservoir and another. (DOCX) [file pone.0261945.s008.docx]

| **Table S1. Results of the PERMANOVA analysis and Post-hoc tests for the evaluation of the Trophic State Index of the study reservoirs, hydrographic basins of the Paraíba and Piranhas-Assú Rivers, Brazil. X corresponds to the test ratio between one reservoir and another.** | | | | | |
| --- | --- | --- | --- | --- | --- |
|  | **DF** | **MS** | **F** | **P-perm** | **Permutations** |
| **Trophic State Index (TSI)** |  |  |  |  |  |
| Reservoirs | 4 | 5692.7 | 62.144 | 0.0001 | 9954 |
| Residual | 130 | 91.605 |  |  |  |
| Total | 334 |  |  |  |  |
| **Post-hoc tests** |  |  |  |  |  |
| **Reservoirs** | **T** | **P-perm** |  |  |  |
| Poções x Sumé | 8.8967 | 0.0001 |  |  |  |
| Poções x Traíras | 1.8223 | 0.0589 |  |  |  |
| Poções x Sabugí | 10.979 | 0.0001 |  |  |  |
| Traíras x Sumé | 8.159 | 0.0001 |  |  |  |
| Traíras x Sabugí | 9.7715 | 0.0001 |  |  |  |
| Sabugí x Sumé | 1.2524 | 0.2152 |  |  |  |
