# Supplementary material for: Perception of the local community: What is their relationship with environmental quality indicators of reservoirs?
Source: PLoS One. 2022 Jan 21;17(1):e0261945. doi: 10.1371/journal.pone.0261945 (PMC8782485; doi:10.1371/journal.pone.0261945)
Supplement: S2 Table — X corresponds to the test ratio between one reservoir and another. (DOCX) [file pone.0261945.s009.docx]

| **Table S2. Results of the PERMANOVA and Post-hoc tests with dissimilarity of the taxonomic benthic macroinvertebrate composition for the study reservoirs, hydrographic basins of the Paraíba and Piranhas-Assú Rivers, Brazil. X corresponds to the test ratio between one reservoir and another.** | | | | | | | | | |
| --- | --- | --- | --- | --- | --- | --- | --- | --- | --- |
| **Source** | **DF** | **MS** | **F** | | | **P-perm** | | **Permutations** | |
| **Taxonomic composition** |  |  |  | | |  | |  | |
| Reservoirs | 4 | 53806 | 38.914 | | | 0.0001 | | 9903 | |
| Residual | 320 | 1382.7 |  | | |  | |  | |
| Total | 324 |  |  | | |  | |  | |
| **Post-hoc tests** |  |  |  | | | | | | |
| **Reservoirs** | **t** | **P-perm** |  |  |  |  |  |  |  |
| Poções x Sumé | 3.672 | 0.0001 |  |  |  | |  | |  |
| Poções x Traíras | 1.2484 | 0.1843 |  |  |  | |  | |  |
| Poções x Sabugí | 8.2304 | 0.0001 |  |  |  | |  | |  |
| Traíras x Sumé | 3.2341 | 0.0001 |  |  |  | |  | |  |
| Traíras x Sabugí | 6.1284 | 0.0001 |  |  |  | |  | |  |
| Sabugí x Sumé | 8.2984 | 0.2152 |  |  |  | |  | |  |
