# Supplementary material for: Perception of the local community: What is their relationship with environmental quality indicators of reservoirs?
Source: PLoS One. 2022 Jan 21;17(1):e0261945. doi: 10.1371/journal.pone.0261945 (PMC8782485; doi:10.1371/journal.pone.0261945)
Supplement: S3 Table — X corresponds to the test ratio between one reservoir and another. (DOCX) [file pone.0261945.s010.docx]

| **Table S3. Results of the PERMANOVA analysis and Post-hoc tests for the anthropogenic influences in the riparian zone, hydrographic basins of the Paraíba and Piranhas-Assú Rivers, Brazil. X corresponds to the test ratio between one reservoir and another.** | | | | | |
| --- | --- | --- | --- | --- | --- |
|  | **DF** | **MS** | **F** | **P-perm** | **Permutations** |
| **Anthropogenic influences in the riparian zone** |  |  |  |  |  |
| Reservoirs | 4 | 20.435 | 12.074 | 0.0001 | 9949 |
| Residual | 330 | 1.6925 |  |  |  |
| Total | 334 |  |  |  |  |
| **Post-hoc tests** |  |  |  |  |  |
| **Reservoirs** | **T** | **P-perm** |  |  |  |
| Poções x Sumé | 0.6040 | 0.879 |  |  |  |
| Poções x Traíras | 4.0582 | 0.001 |  |  |  |
| Poções x Sabugí | 2.1262 | 0.0049 |  |  |  |
| Traíras x Sumé | 4.3198 | 0.0001 |  |  |  |
| Traíras x Sabugí | 5.7404 | 0.0001 |  |  |  |
| Sabugí x Sumé | 2.1894 | 0.0021 |  |  |  |
