# Supplementary material for: Perception of the local community: What is their relationship with environmental quality indicators of reservoirs?
Source: PLoS One. 2022 Jan 21;17(1):e0261945. doi: 10.1371/journal.pone.0261945 (PMC8782485; doi:10.1371/journal.pone.0261945)
Supplement: S4 Table — X corresponds to the test ratio between one local community and another. (DOCX) [file pone.0261945.s011.docx]

| **Table S4. Results of the PERMANOVA analysis and Post-hoc tests for the evaluation of the different conservation perceptions between the study reservoir communities, hydrographic basins of the Paraíba and Piranhas-Assú Rivers, Brazil. X corresponds to the test ratio between one local community and another.** | | | | | |
| --- | --- | --- | --- | --- | --- |
|  | **DF** | **MS** | **F** | **P-perm** | **Permutations** |
| **Conservation Perception Classification** |  |  |  |  |  |
| Reservoirs | 4 | 0.28409 | 5.0007 | 0.0021 | 9949 |
| Residual | 122 | 5.6809 |  |  |  |
| Total | 126 |  |  |  |  |
| Post-hoc tests |  |  |  |  |  |
| **Reservoirs** | **T** | **P-perm** |  |  |  |
| Poções x Sumé | 3.1804 | 0.002 |  |  |  |
| Poções x Traíras | 0.78531 | 0.4303 |  |  |  |
| Poções x Sabugí | 3.2064 | 0.0018 |  |  |  |
| Traíras x Sumé | 2.1928 | 0.0335 |  |  |  |
| Traíras x Sabugí | 2.0814 | 0.0394 |  |  |  |
| Sabugí x Sumé | 0.54226 | 0.6052 |  |  |  |
